# Particulate Matter (PM_10_) Promotes Cell Invasion through Epithelial–Mesenchymal Transition (EMT) by TGF-β Activation in A549 Lung Cells

**DOI:** 10.3390/ijms222312632

**Published:** 2021-11-23

**Authors:** Claudia M. García-Cuellar, Miguel Santibáñez-Andrade, Yolanda I. Chirino, Raúl Quintana-Belmares, Rocío Morales-Bárcenas, Ericka Marel Quezada-Maldonado, Yesennia Sánchez-Pérez

**Affiliations:** 1 Instituto Nacional de Cancerología (INCan), Subdirección de Investigación Básica, San Fernando No. 22, Ciudad de México 14080, Mexico; garcue57@gmail.com (C.M.G.-C.); msantrade@ciencias.unam.mx (M.S.-A.); qbro@hotmail.com (R.Q.-B.); mobarobiol@yahoo.com.mx (R.M.-B.); marelquezada0612@gmail.com (E.M.Q.-M.); 2 Unidad de Biomedicina, Facultad de Estudios Superiores Iztacala, Universidad Nacional Autónoma de México, Los Reyes Iztacala, Tlalnepantla 54090, Estado de México, Mexico; irasemachirino@gmail.com

**Keywords:** PM_10_, *TGFB1*, A549, epithelial–mesenchymal transition, invasion, cancer hallmark

## Abstract

Air pollution presents a major environmental problem, inducing harmful effects on human health. Particulate matter of 10 μm or less in diameter (PM_10_) is considered an important risk factor in lung carcinogenesis. Epithelial–mesenchymal transition (EMT) is a regulatory program capable of inducing invasion and metastasis in cancer. In this study, we demonstrated that PM_10_ treatment induced phosphorylation of SMAD2/3 and upregulation of SMAD4. We also reported that PM_10_ increased the expression and protein levels of *TGFB1* (TGF-β), as well as EMT markers *SNAI1* (Snail), *SNAI2* (Slug), *ZEB1* (ZEB1), *CDH2* (N-cadherin), *ACTA2* (α-SMA), and *VIM* (vimentin) in the lung A549 cell line. Cell exposed to PM_10_ also showed a decrease in the expression of *CDH1* (E-cadherin). We also demonstrated that expression levels of these EMT markers were reduced when cells are transfected with small interfering RNAs (siRNAs) against *TGFB1*. Interestingly, phosphorylation of SMAD2/3 and upregulation of SMAD induced by PM_10_ were not affected by transfection of *TGFB1* siRNAs. Finally, cells treated with PM_10_ exhibited an increase in the capacity of invasiveness because of EMT induction. Our results provide new evidence regarding the effect of PM_10_ in EMT and the acquisition of an invasive phenotype, a hallmark necessary for lung cancer progression.

## 1. Introduction

Air pollution presents one of the major environmental problems, inducing harmful effects in human health. These effects have a major impact in highly populated cities, where industrial activity and vehicle traffic increase emissions of multiple pollutants. Epidemiological and toxicological research led to the classification of air pollution as carcinogenic in Group 1 by the International Agency for Research on Cancer (IARC) [1]. Multiple epidemiologic studies have demonstrated that particulate matter (PM) inhalation has a negative impact on human health, increasing the occurrence of respiratory diseases such as chronic obstructive pulmonary disease, asthma, and, over the long term, lung cancer [2,3,4,5]. According to the International Agency for Research in Cancer (IARC), lung cancer is still the leading cause of cancer death worldwide. In 2018, approximately 1.7 million deaths were attributed to lung cancer cases. Interestingly, from all lung cancer cases, at least 29% were linked with ambient air pollution [6,7]. To act against air pollution, the World Health Organization (WHO) developed the ambient air quality database, with the aim of maintaining particles with an aerodynamic diameter less or equal to 10 µm (PM_10_) and an annual mean value below 20 µg/m^3^. However, the increasing of emissions by industry, power generation, transportation, and domestic burning in highly populated sites exceeds constantly the WHO’s health-based air-quality guidelines.

Epithelial–mesenchymal transition (EMT) is defined as a regulatory program capable of orchestrating the induction of invasion and metastasis processes in cancer, via the loss of the apical-basal polarity in epithelial cells and the acquisition of a mesenchymal phenotype [8,9]. EMT results from certain molecular events, such as the repression of E-cadherin expression, the induction of N-cadherin and vimentin, and the loss of cell–cell junctions [10,11]. These events are accompanied by other cytoskeleton remodeling signals, including the formation of actin stress fibers, and the induction of α-smooth muscle actin (α-SMA) and fibronectin [12].

One of the main initiators for EMT signaling is the transforming growth factor-beta (TGF-β). TGF-β is capable of regulating directly and positively mesenchymal markers such as the alfa-smooth muscle actin (α-SMA) [13]. Additionally, TGF-β acts as a strong regulator of cytoskeleton remodeling [14]. Downstream signaling induced by TGF-β involves the type I and type II TGF-β receptors (TβRI and TβRII) forming a hetero-tetrameric complex by phosphorylation of the TβRI GS domain [15,16,17]. Then, TβRI recruits and phosphorylates two cytoplasmic mediators of TGF-β signaling, the receptor-activated SMAD proteins SMAD2 and SMAD3, which in assistance of SMAD4 are translocated to the nucleus [18].

Although the loss of E-cadherin is the main EMT hallmark, the underlying mechanism for the acquisition of the EMT phenotype include the zinc-finger proteins Snail and Slug and the two-handed zinc-finger protein ZEB1 [19,20]. SMAD2/3/4 as well as other co-activators induce the expression of Snail, Slug, and ZEB1. Then, these transcription factors bind to the promoter region of the human E-cadherin gene (*CDH1*), silencing its transcription [19,21,22].

Cancer progression pathways are activated as a consequence of deregulated expression of genes caused by PM, such as the activation of downstream EMT inducers in lung cancer cells [23,24]. In addition, chronic PM exposure induces EMT morphology [25], different degrees of EMT progression, and increased invasion ability in lung cells in a concentration-dependent manner [26]. Moreover, lung metastasis associated to air pollution has also been associated with the methylation of EMT genes [27].

The effect of PM in migration and invasion pathways has been reported in some in vitro experiments [28]. For example, cells exposed to PM_10_ and PM_2.5_ (particles with an aerodynamic diameter less or equal to 2.5 µm) showed an increase in protease activity, as well as an invasive phenotype, associated with the up-regulation in metalloproteases [29,30,31]. Transepithelial electrical resistance experiments have shown that PM components are capable of increasing cell permeability, enhancing the ability of invasion and orthotopic lung carcinoma metastasis in mice [32]. However, the association between PM exposure, EMT induction, and the acquisition of the invasive phenotype remains unclear.

In the present study, we evaluated the effect of PM_10_ exposure in the induction of TGF-β and the expression/abundance/phosphorylation of EMT components, as well as its impact in the invasive phenotype using the lung epithelial A549 cell line.

## 2. Results

### 2.1. Particulate Matter PM_10_ Increases the Phosphorylation of SMAD2/3 and the Levels of SMAD4 in A549 Cells

**Figure 1:**
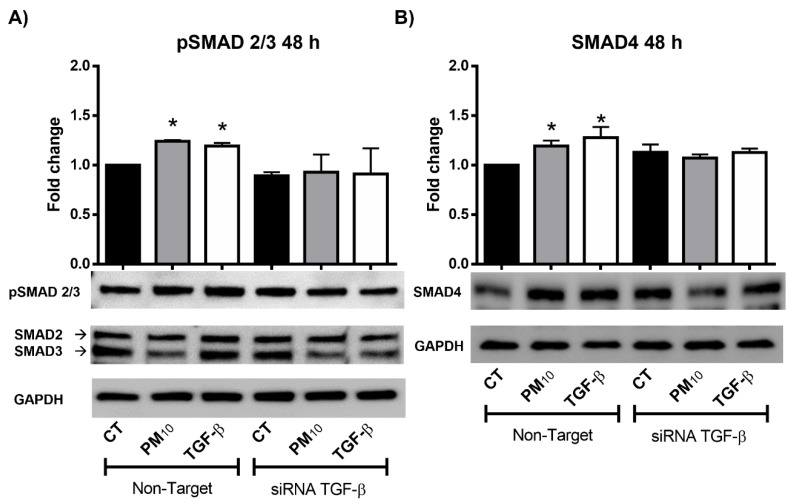
Particulate matter PM_10_ increases the phosphorylation of SMAD2/3 and the levels of SMAD4 in A549 cells after 48 h of treatment: (**A**) Particulate matter exposure increases the phosphorylation levels of SMAD2/3 in A549 cells transfected with non-targeting siRNA, with a similar effect in cells treated with TGF- β for 48 h. (**B**) PM_10_ exposure increases the protein levels of SMAD4 in A549 cells transfected with non-targeting siRNA, with a similar effect in cells treated with TGF- β for 48 h. These effects are not prevented in A549 cells transfected with *TGFB1* siRNAs. Cells were exposed to PM_10_ (10 µg/cm^2^) and TGF-β (5 ng/mL) for 48 h. The quantitative results were expressed after normalization using GAPDH as a loading control. Data are reported as the means ± SD of three independent experiments. The images are representative of the data obtained. (*) indicates statistically differences between treatments. Densitometry was performed using arbitrary units *p* < 0.05.

Cells exposed to PM_10_ for 48 h and transfected with non-targeting siRNA showed an increase in the phosphorylation levels of SMAD2/3 when compared with control group (fold change→FC of 1.24 vs. 1.00; *p* < 0.05) (Figure 1A). In addition, cells treated with TGF-β showed an increase in the phosphorylation levels of SMAD2/3 when compared with control group (FC of 1.19 vs. 1.00; *p* < 0.05). Cells transfected with *TGFB1* siRNAs showed no changes in the phosphorylation levels of SMAD2/3 in non-treated, PM_10_, and TGF-β groups (FC of 0.89, 0.92, and 0.91 vs. 1.00, respectively; *p* < 0.05), suggesting a link between the phosphorylation status of SMAD2/3 and PM_10_ treatment independent of *TGFB1* repression. Moreover, cells exposed to PM_10_ for 48 h and transfected with non-targeting siRNA showed an increase in the protein levels of SMAD4 when compared with control group (FC of 1.19 vs. 1.00; *p* < 0.05) (Figure 1B). Cells treated with TGF-β also showed an increase in protein levels of SMAD4 when compared with control group (FC of 1.27 vs. 1.00; *p* < 0.05). Cells transfected with *TGFB1* siRNAs showed no change in the protein levels of SMAD4 in non-treated, PM_10_, and TGF-β groups (FC of 1.13, 1.07, and 1.12 vs. 1.00, respectively; *p* < 0.05), suggesting a link between the abundance of SMAD4 and PM_10_ treatment independent of *TGFB1* repression.

### 2.2. Particulate Matter PM_10_ Induce the Expression of TGFB1 and EMT Components in A549 Cells

**Figure 2:**
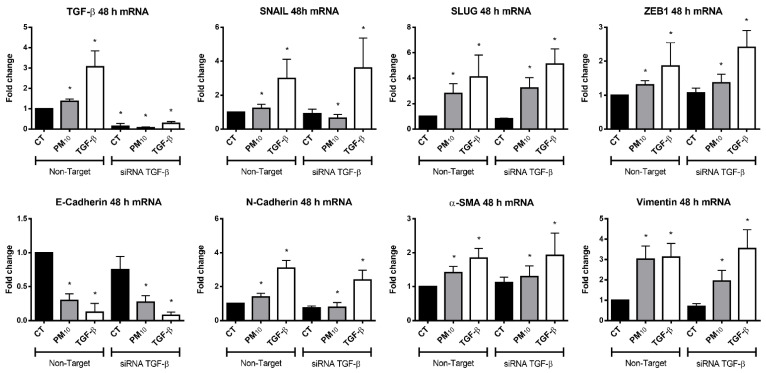
Particulate matter PM_10_ induces the expression of TGFB1 and EMT components in A549 cells after 48 h of treatment. Particulate matter exposure increases the expression levels of TGFB1, *SNAI1* (Snail), *SNAI2* (Slug), *ZEB1* (ZEB1), *CDH2* (N-cadherin) *ACTA2* (α-SMA), and *VIM* (vimentin), and decreases the expression levels of *CDH1* (E-cadherin), while TGF- β treatment increases the expression levels of *TGFB1*, *SNAI1* (Snail), *SNAI2* (Slug), *ZEB1* (ZEB1), *CDH2* (N-cadherin), *ACTA2* (α-SMA), and *VIM* (vimentin), and decreases the expression levels of *CDH1* (E-cadherin) in cultures of A549 cells transfected with non-targeting siRNA. These effects are prevented by *TGFB1* siRNAs only in *TGFB1*, *SNAI1* (Snail), *CDH2* (N-cadherin), and *VIM* (vimentin). Cells were exposed to PM_10_ (10 µg/cm^2^) and TGF-β (5 ng/mL) for 48 h. The quantitative results were expressed after normalization using GAPDH as a control. Data are reported as the means ± SD of three independent experiments. The images are representative of the data obtained. (*) indicates statistically differences between treatments. Relative quantification or fold change (FC) was performed using the 2^−ΔΔCt^; *p* < 0.05.

Cells exposed to PM_10_ for 48 h and transfected with non-targeting siRNA showed an increase in the expression levels of *TGFB1* when compared with control group (FC of 1.36 vs. 1.00; *p* < 0.05) (Figure 2). In addition, cells treated with TGF-β showed an increase in the expression levels of *TGFB1* when compared with control group (FC of 3.06 vs. 1.00; *p* < 0.05). Moreover, cells transfected with *TGFB1* siRNAs showed a decrease in *TGFB1* expression levels in non-treated, PM_10_, and TGF-β groups (FC of 0.13, 0.06, and 0.28 vs. 1.00, respectively; *p* < 0.05), maintaining the effect in *TGFB1* knockdown. Cells exposed to PM_10_ and transfected with non-targeting siRNA also exhibited an increase in expression levels of *SNAI1* (Snail), *SNAI2* (Slug), *ZEB1* (ZEB1), *CDH2* (N-cadherin), *ACTA2* (α-SMA), and *VIM* (vimentin) (FC of 1.21, 2.79, 1.30, 1.39, 1.41, and 3.02 vs. 1.00, respectively; *p* < 0.05), and a decrease in expression levels of *CDH1* (E-cadherin) when compared with control group (FC of 0.29 vs. 1.00, respectively; *p* < 0.05). A similar effect in EMT genes *SNAI1* (Snail), *SNAI2* (Slug), *ZEB1* (ZEB1), *CDH2* (N-cadherin), *ACTA2* (α-SMA), *VIM* (vimentin), and *CDH1* (E-cadherin) was observed in cells treated with TGF-β (FC of 2.98, 4.10, 1.86, 3.09, 1.84, 3.12, and 0.12 vs. 1.00, respectively; *p* < 0.05). When cells were transfected with *TGFB1* siRNAs, EMT gene *SNAI1* (Snail) exhibited a decrease in expression levels in non-treated and PM_10_, but an increase in expression levels in TGF-β group (FC of 0.91, 0.64, and 3.60, respectively; *p* < 0.05) when compared with their non-targeting siRNA pair. A similar effect was observed in *CDH1* (E-cadherin) (FC of 0.75, 0.27, and 0.07, respectively; *p* < 0.05) and *CDH2* (N-cadherin) (FC of 0.75, 0.78, and 2.39, respectively; *p* < 0.05). Cells transfected with *TGFB1* siRNAs exhibited a decrease in expression levels in non-treated group, but an increase in expression levels in PM_10_ and TGF-β-treated groups in *SNAI2* (Slug) (FC of 0.80, 3.22, and 5.11, respectively; *p* < 0.05) and *VIM* (vimentin) (FC of 0.69, 1.94, and 3.54, respectively; *p* < 0.05). On the other hand, cells transfected with *TGFB1* siRNAs exhibited no changes in expression levels in non-treated and PM_10_ groups, but an increase in expression levels in PM10 and TGF-β-treated groups in *ZEB1* (ZEB1) (FC of 1.06, 1.36, and 2.40, respectively; *p* < 0.05) and *ACTA2* (α-SMA) (FC of 1.12, 1.29, and 1.92, respectively; *p* < 0.05).

### 2.3. Particulate Matter PM_10_ Increases the Protein Levels of EMT Markers in A549 Cells

**Figure 3:**
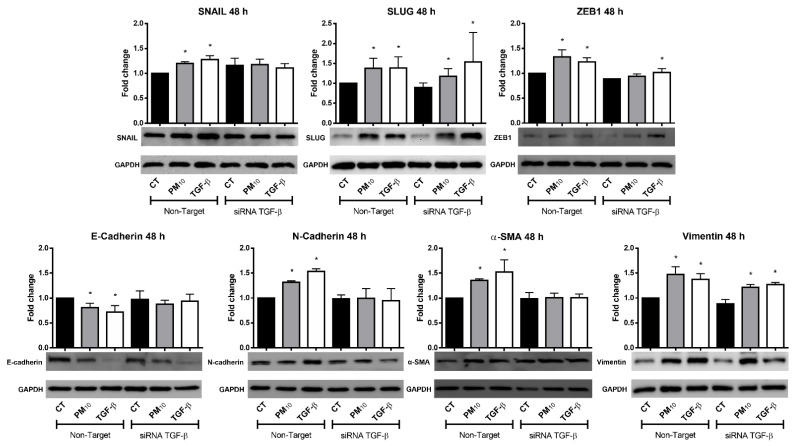
Particulate matter PM_10_ increases the protein levels of EMT markers in A549 cells after 48 h of treatment. Particulate matter exposure increases the protein levels of Snail, Slug, ZEB1, N-cadherin, α-SMA, and vimentin, and decreases the protein levels of E-cadherin, while TGF-β treatment increases the protein levels of Snail, Slug, ZEB1, N-cadherin, α-SMA, and vimentin, and decreases the protein levels of E-cadherin in cultures of A549 cells transfected with non-targeting siRNA. These effects are prevented by *TGFB1* siRNAs only in Slug, ZEB1, N-cadherin, α-SMA, and vimentin. Cells were exposed to PM_10_ (10 µg/cm^2^) and TGF-β (5 ng/mL) for 48 h. The quantitative results were expressed after normalization using GAPDH as a loading control. Data are reported as the means ± SD of three independent experiments. The images are representative of the data obtained. (*) indicates statistically differences between treatments. Densitometry was performed using arbitrary units *p* < 0.05.

Cells exposed to PM_10_ for 48 h and transfected with non-targeting siRNA exhibited an increase in protein levels of *SNAI1* (Snail), *SNAI2* (Slug), *ZEB1* (ZEB1), *CDH2* (N-cadherin), *ACTA2* (α-SMA), and *VIM* (vimentin) (FC of 1.19, 1.38, 1.23, 1.30, 1.35, and 1.47 vs. 1.00, respectively; *p* < 0.05), and a decrease in protein levels of *CDH1* (E-cadherin) when compared with the control group (FC of 0.80 vs. 1.00, respectively; *p* < 0.05) (Figure 3). A similar effect in EMT markers was observed in cells treated with TGF- β, with an increase in protein levels of *SNAI1* (Snail), *SNAI2* (Slug), *ZEB1* (ZEB1), *CDH2* (N-cadherin), *ACTA2* (α-SMA), and *VIM* (vimentin) (FC of 1.27, 1.38, 1.23, 1.53, 1.52, and 1.37 vs. 1.00, respectively; *p* < 0.05) and a decrease in protein levels of *CDH1* (E-cadherin) (FC of 0.72 vs. 1.00, respectively; *p* < 0.05). When cells were transfected with *TGFB1* siRNAs, EMT marker *SNAI1* (Snail) exhibited an increase in protein levels in non-treated, PM_10_, and TGF-β groups (FC of 1.15, 1.17, and 1.10, respectively; *p* < 0.05) when compared with their non-targeting siRNA pair. *SNAI2* (Slug) exhibited a decrease in protein levels in the non-treated group, followed by an increase in PM_10_-treated and TGF-β groups (FC of 0.89, 1.18, and 1.53, respectively; *p* < 0.05). *ZEB1* (ZEB1) exhibited a decrease in protein levels in non-treated and PM_10_ groups, with no changes in TGF-β group (FC of 0.88, 0.93, and 1.01, respectively; *p* < 0.05). *CDH1* (E-cadherin) exhibited no changes in protein levels in the non-treated group, followed by a decrease in PM_10_-treated group and no changes in TGF-β group (FC of 0.97, 0.87, and 0.93, respectively; *p* < 0.05). *CDH2* (N-cadherin) exhibited no changes in protein levels in the non-treated, PM_10_-treated, and TGF-β groups (FC of 0.98, 0.99, and 0.94, respectively; *p* < 0.05). *ACTA2* exhibited no changes in protein levels in the non-treated, PM_10_-treated, and TGF-β groups (α-SMA) (FC of 0.98, 1.00, and 1.00, respectively; *p* < 0.05). Finally, *VIM* (vimentin) exhibited a decrease in protein levels in the non-treated group, followed by an increase in PM_10_-treated and TGF-β groups (FC of 0.88, 1.21, and 1.26, respectively; *p* < 0.05).

### 2.4. Particulate Matter PM_10_ Activates the Invasive Phenotype in A549 Cells

**Figure 4:**
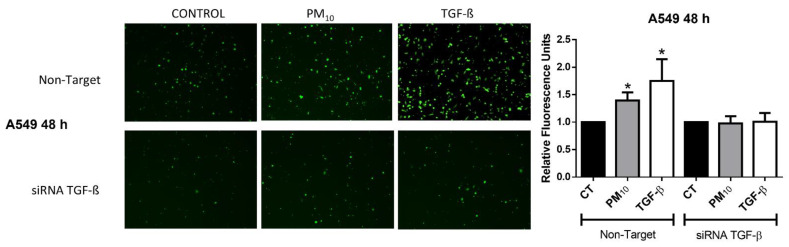
Particulate matter PM_10_ activates the invasive phenotype in A549 cells. Particulate matter exposure increases the invasiveness of A549 cells exposed to PM_10_ and transfected with non-targeting siRNA, with a similar effect in cells treated with TGF-β for 48 h. These effects are prevented in A549 cells transfected with *TGFB1* siRNAs. Cells were exposed to PM_10_ (10 µg/cm^2^) and TGF-β (5 ng/mL) for 48 h. The quantitative results were expressed after obtaining the relative fluorescence units of each condition. Data are reported as the means ± SD of three independent experiments. The images are representative of data obtained. (*) indicates statistical differences between treatments *p* < 0.05.

A549 cells exposed to PM_10_ for 48 h and transfected with non-targeting siRNA showed an increase in the invasiveness levels when compared with control group (FC of 1.39 vs. 1.00; *p* < 0.05) (Figure 4). In addition, cells treated with TGF-β showed an increase in invasiveness level when compared with control group (FC of 1.74 vs. 1.00; *p* < 0.05). Cells transfected with *TGFB1* siRNAs showed no changes in invasiveness levels in the non-treated, PM_10_, and TGF-β groups (FC of 1.00, 0.97, and 1.00 vs. 1.00, respectively; *p* < 0.05), suggesting a role of *TGFB1* expression in the induction of the invasive phenotype.

## 3. Discussion

In the present study, we evaluated the effect of PM_10_ in the induction of EMT through the TGF-β pathway, considering the phosphorylation of EMT cascade initiators such as SMAD2/3, as well as the upregulation of SMAD4, in order to follow the transcription activation of multiple EMT markers. We found that PM_10_ is capable of inducing the expression of *TGFB1* and EMT markers *SNAI1*, *SNAI2*, *ZEB1*, *CDH2*, *ACTA2,* and *VIM* as well as their respective products TGF-β, Snail, Slug, ZEB1, N-cadherin, α-SMA, and vimentin in the lung A549 cell line. Cell exposed to PM_10_ also shows a decrease in the expression of *CDH1* and its product, E-cadherin. Interestingly, expression levels of these EMT markers were reduced when cells are transfected with small interfering RNAs (siRNAs) against *TGFB1*. However, phosphorylation of SMAD2/3 and upregulation of SMAD induced by PM_10_ was not affected by transfection of *TGFB1* siRNAs. As a response to these molecular changes in EMT components on cells treated with PM_10_, the biological consequence is the increase in the capacity of invasiveness.

EMT is involved in multiple pulmonary fibrotic diseases as well as in lung cancer [12,33,34,35,36]. According to the physiological and pathological processes where is involved, EMT has been classified into three types. While type 1 and type 2 are related to embryonic development and tissue regeneration/cicatrization, respectively, type 3 EMT has been widely recognized as necessary for cancer cells in order to confer an invasive and metastatic phenotype, where cancer cells not only show conversion from epithelial to mesenchymal cells, but also undergo mesenchymal–epithelial transition (MET) in sites where they are established after metastasis [37]. Some studies have detected the induction of type 2 and 3 EMT in lung diseases, providing some clues regarding the relevance of PM in EMT [38,39].

Cancer pathways where EMT is involved are well known. In this context, the induction of EMT is mainly associated with factors such as TGF-β, SMAD2/3, MAPK, IL-6, Wnt, SHH, and Notch [40,41]. The target of these pathways involves multiple transcription factors but show affinity to *SNAI1*, *SNAI2,* and *ZEB*. This step in the EMT signaling is crucial because these transcription factors have a direct impact on E-cadherin expression. Thus, EMT signaling is oriented to cause an inhibition of epithelial markers and, in the same way, cause an induction of mesenchymal markers.

EMT markers that are well established and usually detected in vitro or in vivo include a decrease in E-cadherin expression, accompanied by an increase in the expression levels of N-cadherin, α-SMA, and vimentin [41,42]. Additionally, an increase in the expression levels of MMPs and type 1 collagen is also induced [43,44]. Together, these EMT components create a microenvironment in which growth factors, degrade extracellular matrix enzymes and extracellular matrix proteins, and orchestrate the activation of autocrine and paracrine signals oriented to sustain EMT in cancer [45,46].

The mechanisms by which air pollution is capable of inducing cell transformation depend on a complex interaction between environmental and individual factors. PM is a mixture of organic, inorganic, and biological entities interacting not only in the air, but also in their site of deposition. The composition of this mixture is dependent of the location where the PM is collected, the sources of emission near to the collection site, and the season in which PM is sampled [47,48,49,50]. While epidemiological studies have reported a sustained increase in risk for lung cancer incidence of approximately 8% per each increase of 10 µg/m^3^ in PM_10_ concentrations [51], other evidence points to the fact that PM_10_ as well as PM_2.5_ are associated with poorer lung cancer survival, emphasizing their effect in early stage non-small cell lung cancer [52]. Because air pollution has been catalogued as a Group I carcinogen by the IARC, the underlying process of malignant cell transformation is expected to follow the multistep sequence described for these group of diseases: initiation, promotion, and progression [53]. As part of the cancer progression step, a reversible process called epithelial–mesenchymal transition (EMT) takes place. The goal of EMT in cancer is the colonization of distant sites, starting with the signaling necessary for the induction of a phenotype suitable for cell migration (mesenchymal), followed by the invasion of adjacent tissues, and finally, colonizing distant tissues trough metastasis and promoting the establishment by switching again the phenotype through the epithelial condition [54]. Thus, the EMT process is the program that converts a benign tumor into an aggressive and highly invasive neoplasm.

Several cancer progression programs are subject to change by PM_10_, and these effects have been linked to deregulated expression of genes caused by PAHs contained in PM [24]. Further, PM exposure activates inducers of EMT in lung cancer cells, impacting positively the downstream signaling of multiple mesenchymal markers [23]. Chronic exposure to PM also induces a well-defined EMT morphology in A549 cells [25]. Furthermore, chronic exposure has described the carcinogenic mechanisms of PM in the induction of EMT, emphasizing the role of the organic fraction in the induction of different degrees of EMT progression and increased invasion ability in BEAS-2B cells in a concentration-dependent manner [26]. In fact, BEAS2B exposed to PM exhibits a decrease in E-cadherin, followed by an increase in mesenchymal markers such as α-SMA, Snail, Slug, ZEB1, and extracellular matrix degradation related to an increase in TGFB1, MMP3, and MMP9 [55].

The effect of PM in migration and invasion pathways in cancer cells has also been described in A549 cells. Toxicological assays as well as global transcriptome profiling exhibit an increase in the activity of proteases, as well as an invasive phenotype (due to the up-regulation of metalloproteases) when cells are exposed to PM_10_ and PM_2.5_ [28,29,30,31]. PM exposure has been also associated with methylation of EMT genes [27]. PM components also increase cell permeability, according to experiments measuring transepithelial electrical resistance. Deregulation of metalloprotease activity, as well as other signaling pathways, enhance the ability of invasion and induce orthotopic lung carcinoma metastasis in mice [32]. This evidence suggests that both, PM_10_ and PM_2.5_ are potent signal transducers, acting as activators of transcription of key regulators in EMT, a condition strongly linked to tumor progression [56].

A variety of cell lines have been considered for the evaluation of the effect of PM in the induction of EMT. These models include the epithelial bronchial cell lines BEAS2B, 16HBE, HBEC, and HBEC3, as well as the alveolar carcinoma cell line A549 and the lung carcinoma NCI-H441 and H292 cell lines. From these, BEAS2B and A549 are the most frequently used models [57]. Although several studies report similar effects of PM in both cell lines, we considered the A549 cell line to be a more suitable model for the study of EMT, once that is a transformed cell line. This feature is crucial in the evaluation of the carcinogenic process because invasion and metastasis are cancer hallmarks involved in progression. We performed experiments in A549 cells treated with PM_10_ for 24 h (data not shown). However, the effects of PM_10_ in EMT are more evident after 48 h of exposure. Moreover, when we performed the invasion assay in BEAS2B, no changes were observed when cells were treated with PM_10_ or TGF-β and compared with controls (data not shown). These results suggest that BEAS2B could lack of some cancer hallmarks necessary for the acquisition of an invasive phenotype. Further studies should be addressed in search of the invasion capacity between these models in order to consider if a non-transformed cell line is suitable for analysis of EMT.

**Figure 5:**
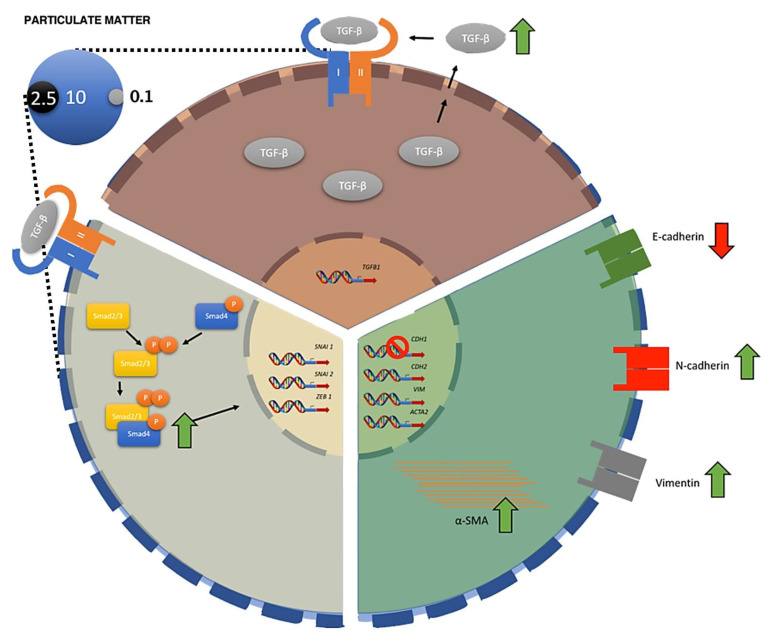
Schematic representation of the effect of PM_10_ in EMT. PM_10_ exposure cause overexpression of *TGFB1* in A549 cells, promoting the autoinduction of TGF-β signaling. As a result, *SNAI1* (Snail), *SNAI2* (Slug), *ZEB1* (ZEB1), *CDH2* (N-cadherin), *ACTA2* (α-SMA), and *VIM* (vimentin) genes are overexpressed, while *CDH1* (E-cadherin) is downregulated in a *TGFB1*-dependent way. PM_10_ also induces phosphorylation of SMAD2/3 and an increase in protein levels of SMAD4 in a *TGFB1*-independent way. These alterations predispose cells to the acquisition of an invasive phenotype, as evidenced by the invasion assay. Stop signal (in CDH1) represents gene repression, while green and red arrows represent increase and decrease of protein levels, respectively.

To our knowledge, this is the first study that has demonstrated the effect of PM_10_ on the induction of the growth factor TGF-β, and its influence on EMT regulation. PM_10_ induces *TGFB1* overexpression followed by overexpression of EMT genes *SNAI1* (Snail), *SNAI2* (Slug), *ZEB1* (ZEB1), *CDH2* (N-cadherin), *ACTA2* (α-SMA), and *VIM* (vimentin), and a decrease in the expression of *CDH1* (E-cadherin) (Figure 5). These changes in gene expression result in TGF-β autoinduction, the activation of type 3 EMT, and the subsequent acquisition of an invasive phenotype, features observed in cancer cells. We also demonstrated that PM_10_ is capable of inducing phosphorylation of SMAD2/3 and an increase in protein levels of SMAD4 in A549 cells treated with PM_10_. Our results suggest that phosphorylation of SMAD2/3, as well as an increase in levels of SMAD4, are not influenced by *TGFB1* overexpression caused by PM_10_ exposure in A549 cells. Moreover, this is the first in vitro study to document the invasive phenotype associated with PM_10_ exposure, integrating gene expression, protein synthesis, and signal transduction of multiple EMT markers. These findings offer new evidence regarding the effect of air pollution in tumor progression, by the acquisition of a key hallmark in carcinogenesis. The effect of air pollution in EMT signaling needs to be evaluated by other approaches, such as the analysis of EMT markers in vivo exposed to PM or of lung cancer patients exposed to air pollution, to determine the role of this risk factor during cancer continuum, and to associate the effect of PM_10_ in lung cancer susceptibility, incidence, and survival.

## 4. Materials and Methods

### 4.1. PM_10_ Sampling

Collection of PM_10_ was performed in Mexico City area using the GMW model 1200 VFC HVPM10 high-volume particle collector with a flux of 1.13 m^3^/min (Sierra Andersen, Atlanta, GA, USA). Particles were captured on cellulose nitrate filters with a pore size of 3.0 μm (Sartorius AG, Goettingen, Germany) for 3 d per week. Storage of the filters was made at 4 °C in dark and dry conditions. After that, particles were removed by gently scraping off membranes, using a surgical blade. Samples were placed for storage into endotoxin-free glass vials. All vials were maintained at 4 °C in dark and dry conditions. Prior to their use for treatment in cell cultures, particles were always sterilized for 15 min at 121 °C and 15 PSI. Characterization of collected PM_10_ is available in previous reports [58,59,60].

### 4.2. Lung Epithelial A549 Cell Culture and Exposure

Cell line A549 (human adenocarcinoma alveolar basal epithelial cells) was obtained from the American Type Culture Collection (ATCC, Manassas, VA, USA). Cell culture was performed using F-12 Kaighn’s medium (Gibco, New York, NY, USA) at 37 °C under an atmosphere of 5% of CO_2_. Only cultures reaching a confluence of 80% of the surface were considered before propagation or experiment running. All experiments were performed in 6-well plates, considering an initial concentration of 250,000 cells per well. Cells were kept in F-12 Kaighn’s medium considering a concentration of 10% of fetal bovine serum (FBS) (Gibco, Brooklyn, NY, USA) for 48 h. Before treatment (4 h prior to exposure), cells were transfected with the Silencer Select Negative Control small interfering RNA (siRNA) (Ambion-Thermo Fisher, Waltham, MA, USA), a control non-targeting siRNA, employing the Xfect RNA Transfection Reagent (Takara, Clontech, Beijing, China) according to manufacturer’s protocol. A549 cells were exposed to non-targeting siRNA to a final concentration of 20 nM. After 4 h, medium was removed, plates were then washed with PBS, and cells were treated with PM_10_, considering a final concentration of 10 µg/cm^2^ in F-12 Kaighn’s medium supplemented with 10% of FBS. This dose is equivalent to a 5-day exposure in humans, according to previous reports [61,62]. Cultures were evaluated at 48 h of treatment, comparing results with control cells (cultures without PM_10_ treatment) Additionally, experiments included cells treated for 48 h with TGF-β, 5 ng/mL (Sigma, St. Louis, MI, USA), serving as positive control of EMT induction.

### 4.3. Knockdown of TGF-β

To compare the effect of PM_10_ in the induction of EMT markers and evaluate the endpoints proposed, cultures of A549 cells were also transfected using a mixture of small interference RNAs (siRNAs) against *TGFβ1* (mRNA of *TGF-β)*, targeting multiple regions of its mRNA to improve the likelihood of degradation. Assays were performed in 6-well plates using an initial concentration of 250,000 cells per well. Cells were kept in F-12 Kaighn’s medium with 10% of fetal bovine serum (FBS) (Gibco, Brooklyn, NY, USA) for 48 h. Then, medium was removed, plates were washed in PBS and cells were synchronized by serum starvation for 24 h using free-FBS F-12 Kaighn’s medium. Before treatment (4 h prior to exposure), cells were transfected with the *TGFβ1* siRNA (h2) (sc-270322) (Santa Cruz Biotechnology, Paso Robles, CA, USA), employing the XfectTM RNA Transfection Reagent (Takara, Clontech, Beijing, China) according to manufacturer’s protocol. A549 cells were exposed to *TGFβ1* siRNA to a final concentration of 20 nM (Supplementary Figure S1). After 4 h, medium was removed, plates were then washed in PBS, and cells were treated with PM_10_ (10 µg/cm^2^), and TGF-β (100 ng/mL; Sigma, St. Louis, MI, USA), respectively. Comparisons in all experiments were performed considering two conditions (cells transfected with non-targeting siRNA and cells transfected with *TGFβ1* siRNAs) with three experimental groups each of them (control/non-treated, PM_10_, and TGF-β).

### 4.4. Western Blot Analysis

Levels of EMT proteins were analyzed by Western blotting. Cell cultures were exposed to PM_10_ (10 µg/cm^2^) for 48 h. Then, cells were washed with PBS and lysed for 5 min in RIPA buffer (20 mM Tris-HCl pH 8; 150 mM NaCl; 1% NP-40) together with a protease inhibitor cocktail (Roche, Mannheim, Germany). Then, lysates were centrifuged at 12,000 rpm and 4 °C for 5 min and supernatants were collected. Protein concentration was analyzed using the bicinchoninic acid assay (Sigma, MI, USA). For each treatment, 20 µg of protein extract was separated by SDS–PAGE for SMAD2/3, pSMAD2 (Ser465/467), pSMAD3 (Ser423/425), SMAD4, Snail, Slug, ZEB1, E-cadherin, N-cadherin, α-SMA, and vimentin and transferred to polyvinylidene difluoride (PVDF) membranes. Membranes were kept in agitation with 5% low-fat milk—0.1% Tween 20 in TBS blocking buffer at room temperature for 1 h. Membranes were incubated with primary antibody (anti-SMAD2/3 (5678), dilution 1:1000; anti-pSMAD2 (Ser465/467)/pSMAD3 (Ser423/425) (8828), dilution 1:1000; anti-SMAD4 (46535), dilution 1:1000; anti-Snail (sc-271977), dilution 1:500; anti-Slug (9585), dilution 1:500, anti-ZEB1 (3396S), dilution 1:1000; anti-E-cadherin (MAB3199), dilution 1:1000; anti-N-cadherin (ab98952), dilution 1:1000; anti-α-SMA (19245S), dilution 1:1000; anti-vimentin (ab922547), dilution 1:5000) (Cell Signaling, Danvers, MA, USA; Santa Cruz Biotechnology, CA, USA; MilliporeSigma, MA, USA; Abcam, Cambrindge, UK) overnight at 4° C using GAPDH as control protein (anti-GAPDH, dilution 1:3000) (Santa Cruz Biotechnology, Dallas, TX, USA). Membranes were washed by agitation in 0.1% Tween 20 in TBS and incubated with HRP-linked secondary antibodies (anti-rabbit IgG, dilution 1:3000). Finally, membranes were incubated with chemiluminescent peroxidase substrate (MilliporeSigma, Burlington, MA, USA). An UVP luminescence reader was used to visualize the results and densitometry analysis was performed in the ImageJ software. Data were normalized using the GAPDH protein levels, and results were presented as the fold change over the control.

### 4.5. RNA Isolation and Expression Analysis

Total RNA isolation from A549 cells was performed using Trizol Reagent (Invitrogen, Waltham, MA, USA) after 48 h of exposure to PM_10._ RNA quantification was obtained using the ND-1000 spectrophotometer (NanoDrop Technologies, Wilmington, MA, USA), with samples having a purity (A260/A280 absorbance ratio) between 1.8 and 2. cDNA synthesis was performed using the High-Capacity cDNA Reverse Transcription Kit (Applied Biosystems, Thermo Fisher Scientific, Lithuania) according to the manufacturer’s specifications. Primers for the evaluation of expression analysis included *TGFB1 (TGF-β)* (forward primer [fwd] 5′-TGAAGCAATAGTTGGTGTCCA-3′ and reverse primer [rev] 5′-GCAGGGATAACACACTGCAA-3′) and the following EMT genes: *SNAI1* (Snail) (fwd 5′-TTCTCACTGCCATGGAATTCC’-3′ and rev 5′-GCAGAGGACACAGAACCAGAAA-3′), *SNAI2* (Slug) fwd 5′-AGATGCATATTCGGACCCAC-3′ and rev 5′-CCTCATGTTTGTGCAGGAGA-3′), *ZEB1* (ZEB1) (fwd 5′-GCACCTGAAGAGGACCAGAG-3′ and rev 5′-TGCATCTGGTGTTCCATTTT-3′), *CDH1* (E-cadherin) (fwd 5′-CAACGACCCAACCCAAGAA-3′ and rev 5′-CCGAAGAAACAGCAAGAGCA-3′), *CDH2* (N-cadherin) (fwd 5′-AAAGAACGCCAGGCCAAAC-3′ and rev 5′-GGCATCAGGCTCCACAGTGT-3′), *ACTA2* (α-SMA) (fwd 5′-CAGCACCGCCTGGATAGCC-3′ and rev 5′-AGGCACCCCTGAACCCGAA-3′) and *VIM* (vimentin) (fwd 5′-CGTCTCTGGCACGTCTTGAC-3′ and rev 5′-GCTTGGAAACATCCACATCGA-3′). RT- qPCR reactions were performed using the qPCR SYBR Kit (Takara, Clontech, China) according to the manufacturer’s protocol, using the StepOne plus Real-Time PCR System (Applied Biosystems, Thermo Fisher Scientific, Waltham, MA, USA). The relative quantification or fold change (FC) was obtained using the 2^−ΔΔCt^ method, considering GAPDH as internal control.

### 4.6. Cell Invasion Assay

Invasion capacity was evaluated using a mobility system according to manufacturer protocol (BioCoat BD, Franklin Lakes, NJ, USA). After treatment with PM_10_, 50,000 cells were seeded in a mobile, 24-well chamber previously rehydrated. Then, 750 µL of chemoattractant (DMEM with 5% FBS) was added to the lower chamber, and samples were incubated for 22 h at 37 °C under an atmosphere of 5% of CO_2_. After that, medium was removed from the apical compartment, and the chamber was transferred to a second mobile plate of 24 wells containing 500 µL per well of calcein AM (4µg/mL in Hank’s balanced salt solution (HBSS) and incubated for 1 h at 37 °C under an atmosphere of 5% of CO_2_. Fluorescence of invaded cells was analyzed in a fluorescence plate reader (Tecan, GENios Plus, Männedorf, Switzerland) with bottom reading capabilities at excitation/emission wavelengths of 485/530 nm without further manipulation. Only those labeled cells that invaded the Matrigel Matrix and passed through the pores of the FluoroBlok membrane were detected using an inverted fluorescence microscope (40×, Carl Zeiss, AxioVert 200 M, Zeiss, Oberkoche, Germany).

### 4.7. Statistical Analysis

Gene expression, protein levels/phosphorylation, and invasion assays were analyzed by one-way analysis of variance and *post hoc* comparisons performed with Bonferroni’s and Dunn’s tests. Analysis was performed in the GraphPad Prism 6 software. Statistically significant differences were considered at the 95% level (*p* < 0.05).

## 5. Conclusions

PM_10_ induces overexpression of *TGFB1* and genes involved in the activation of the epithelial–mesenchymal transition in lung cancer cell line A549, predisposing cells to the acquisition of an invasive phenotype, a hallmark necessary during the carcinogenic process.

## Data Availability

The data that support the findings of this study are available from the corresponding author upon reasonable request.

## Supplementary Materials

The following are online at https://www.mdpi.com/article/10.3390/ijms222312632/s1.
