# Supplementary material for: Particulate Matter (PM10) Promotes Cell Invasion through Epithelial–Mesenchymal Transition (EMT) by TGF-β Activation in A549 Lung Cells
Source: Int J Mol Sci. 2021 Nov 23;22(23):12632. doi: 10.3390/ijms222312632 (PMC8657922; doi:10.3390/ijms222312632)
Supplement: Supplementary file 1 [file ijms-22-12632-s001.zip › ijms-1461623-supplementary.pdf]

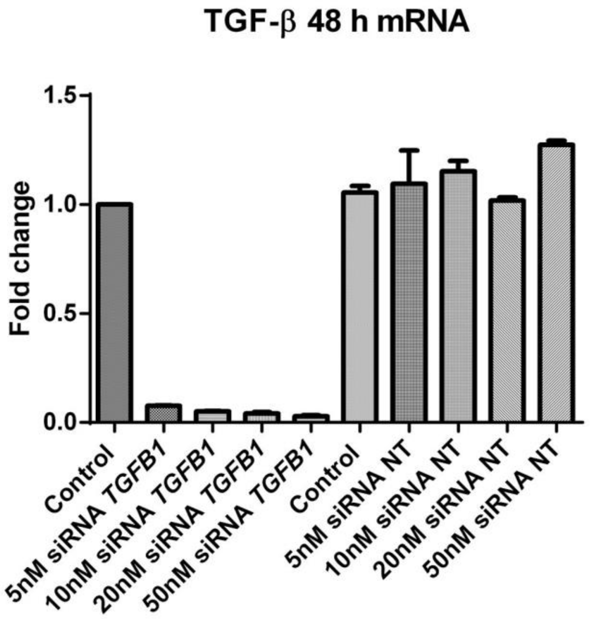

Supplementary Figure S1. Dose/response curve of siRNA TGFB1 and non-targeting siRNA in A549 cells after 48 h of transfection. Silencing of TGF-β mRNA by siRNA TGFB1 is observed since 5nM to 50 nM, while non-targeting siRNA has no effect in TGF-β mRNA silencing at the same doses. The quantitative results were expressed after normalization using GAPDH as a control. Silencing of TGFB1 after siRNA TGFB1 treatment was: 92% for 5 nM; 95% for 10nM; 96% for 20nM and 97% for 50 nM. Data are reported as the means ± SD of three in-dependent experiments. The images are representative of the data obtained. (*) indicates statistically differences between treatments, while (#) indicates statistically differences between similar treatments of both groups. Relative quantification or fold change (FC) was performed using the $2^{-\Delta\Delta Ct}$; $p<0.05$.
